# Editorial: Application of lung ultrasound in the management of pediatric lung diseases

**DOI:** 10.3389/fped.2023.1140403

**Published:** 2023-01-25

**Authors:** Jing Liu, Jovan Lovrenski, Francesco Feletti

**Affiliations:** ^1^ Department of Neonatology and NICU, Beijing Chao-Yang Hospital, Capital Medical University, Beijing, China; ^2^ Radiology Department, Faculty of Medicine, Institute for Children and Adolescents Health Care of Vojvodina, University of Novi Sad, Novi Sad, Serbia; ^3^ Unit of Radiology, Ospedale S. Maria Delle Croci Ravenna, Ausl Romagna, Ravenna, Italy; ^4^ Department of Translational Medicine and for Romagna, University of Ferrara, Ferrara, Italy

**Keywords:** diagnostic imaging, pneumonia, anaesthesia, decision-making, lung ultrasound, lung disease

**Editorial on the Research Topic** Application of lung ultrasound in the management of pediatric lung diseases

## Thirteen years of pediatric lung ultrasound

Lung ultrasound (LUS) has probably been underutilised in paediatrics; however, despite alternating phases of interest, its indications have expanded over time.

Since 2010, LUS has been introduced in paediatrics to assess the opacities detected by CXR.

For example, it was proposed to examine the radiological finding of a white hemithorax.

LUS was exploited for the differential diagnosis between lung pathology and conditions affecting the pleura, mediastinum and thoracic wall.

However, until 2015 more attention was paid to the limitations than the method's advantages. The most critical limitations were

• the lack of studies on pediatric pathology,
• the need for an adequate acoustic window to visualise lung pathology,
• the indirect nature of the assessment of the lung based on acoustic artefacts, and
• the contrasting opinions of the different authors regarding the method.

Since 2018, new studies aimed at defining specific ultrasound patterns and ultrasound score specific for the various pediatric lung diseases in specific clinical settings. As a result, it was fostered the use of LUS as a non-invasive clinical marker to monitor the evolution and treatment of many lung diseases (1).

The covid epidemic, starting in 2019, has helped to give new impetus to the use of the method thanks to the possibility of obtaining information of practical use quickly, with little human contact and easily sanitised equipment.

There is an ongoing growth in scientific interest in the clinical use of Lung Ultrasound (LUS) in pediatry. A PubMed search [using the following string: (pediatr* AND “lung ultrasound”)] from January 2013 to January 2023 results in 503 results. Among them, 79.1% (*n* = 398) were published from 2020, showing a trend in increase and 138 results only in 2022. This attention of the scientific literature reflects that LUS has recently become a frequently used diagnostic tool in pediatric clinical practice among clinicians and radiologists.

LUS can significantly reduce the use of chest x-rays (CXRs) in the pediatric population, from diagnosing pediatric pneumonia to its application in neonatal intensive care.

The enthusiasm for LUS depends on the fact that it is a commonly available, user-friendly, cheap imaging technique with no ionising radiation. LUS is complementary to bedside CXR and can reduce the need for CT scans. In addition, LUS allows the assessment of pleural conditions that may be associated with lung conditions or should be differentiated by lung pathologies, including pleural effusion and pneumothorax.

## The technique of execution of lung ultrasound in pediatry

LUS encounters two unique physical obstacles: alveolar air and the structures of the chest wall, rib cage included. However, these limitations are reduced when studying pediatric chest LUS due to the chest wall's reduced subcutaneous fat and partial ossification. The thymus also represents a supplementary acoustic window for evaluating the anterior chest.

LUS exploits 3–5 MHz convex transducers or above 10 MHz linear arrays depending on the age of the patient and the size of the thorax to be examined. Small high-frequency linear probes are preferred in neonates and infants where the thorax is mainly assessed through sagittal and intercostal scans. The lung shall be explored extensively through longitudinal, transversal, and intercoastal scans. Studying the lung with ultrasound requires proceeding systematically: generally from top to bottom in the ventral-dorsal direction, using parasternal, medial clavicular, and axillary lines as benchmarks. When the patient can keep seated, probes should also be placed along interscapular and paravertebral lines. An upper clavicular approach assures the examination of the lung apices. Finally, the liver and the spleen's subcostal and intercostal acoustic windows are used to study the lung base. A standard scanning protocol identifies 12 lung areas, six for each hemithorax. The areas are delimitated by anterior and posterior longitudinal axillary lines and two axial lines: one about 1 cm above the nipples and the further above the diaphragm. Therefore two anterior areas are identified between the sternum and the anterior axillary line, two lateral between the axillary lines, and the posterior between the posterior axillary line and the spine (1, 2).

## Imaging findings and semeiotics

The pleura interface appears as a distinctly hyperechogenic line, where lung sliding, the movement due to the expansion of the lung, can be identified during breathing. Since ultrasounds cannot penetrate air-filled anatomical structures, normal lung parenchyma is not visible. Instead, when under the pleura, the air creates a strong ultrasound reflection, and linear reverberation artefacts parallel to the pleural line called the “A lines” are visible. A-lines are present in normal lungs and the case of pneumothorax. In pneumothorax, however, the lung sliding is missing as the visceral pleura is separated from the parietal pleura. The lung point is where the two layers of the pleura contact and the lung sliding reappear. In pathological conditions, peripheric lung tissue with reduced aeration that forms acoustic traps ultimately acting as secondary US sources produces vertical artefacts (B-lines). Crucially, these artifactual phenomena depend highly on the US frequency (3). Losing alveolar air permits ultrasound penetration and direct visualisation of the lung parenchyma immediately below the pleura or through the acoustic window created by pleural effusion. Therefore, both atelectasis and consolidative pneumonia appear hypoechogenic, more or less homogeneous. In pneumonia, the reverberant linear images of the air bronchogram have a branch-shaped aspect.

On the contrary, in atelectasis, whenever present, they have a parallel distribution. Sonography can detect even minimal pleural effusion (about 5 ml), which is not evident on a CXR (4). Transudates are usually anechoic, while exudates may be corpuscular and present septae quickly demonstrated sonographically.

## Typical applications of lung ultrasound in pediatry

The World Health Organization (5) reported that pneumonia killed 740,180 children in 2019, accounting for 14% of all deaths of children under five. For the diagnosis of pediatric pneumonia, LUS has a sensitivity (95.0%) and specificity (96.1%) (6) higher than a chest x-ray and is now considered as one of the preferable first-line modality tests (7).

LUS may also be more sensitive and specific than CXR to identify pulmonary fluid overload (8). Moreover, perioperative LUS can identify signs of pulmonary over circulation, such as pulmonary oedema and pleural fluid in children with congenital heart disease, with sensitivity and specificity of lung ultrasound (96% and 95%, respectively) similar to chest CT and far superior to CXR (74% and 50%, respectively) (9–12).

In Critically ill Children, fluid overload is a common complication associated with increased mortality and morbidity. Specifically, this condition may affect young patients after bone marrow transplantation or congenital heart surgery, as well as those suffering from chronic kidney disease and severe sepsis. LUS is frequently used in pediatric anaesthesia to guide airway management and assess one-lung ventilation, especially in respiratory or hemodynamic compromise (11, 12). Ultrasound-guided lung recruitment manoeuvres have been proposed in pediatric and adolescent patients both for managing acute respiratory distress syndrome since 2009 (13) and to optimise mechanical ventilation in children undergoing congenital heart surgery (14). When lung surgery requires lung separation, LUS can confirm the correct lung is being ventilated and the absence of lung sliding on the non-ventilated side; however it is not optimal for determining which lobes are being ventilated (12, 15).

Interstitial lung disease is a common manifestation in autoimmune and connective tissue diseases, systemic sclerosis included. There is evidence of utilising LUS in screening interstitial lung disease, even in the earlier stages of the condition and when lung involvement is subclinical. Moreover, the existing data suggest that LUS can support clinical evaluation during therapeutic follow-up (16).

LUS has been reported as a valuable tool for assessing lung injuries in pediatric trauma. Slight lung contusions may appear as B-lines and can be monitored, avoiding recourse to radiology. Even in more severe cases, LUS was used instead of radiology to follow up lung injuries, including post-traumatic hemato-pneumatocele, allowing minimisation of x-ray exposure (17).

In the radiology departments, LUS is a complementary investigation of CRX, able to clarify the nature of radiological opacification of the lung, even in the case of a white hemithorax. Indeed LUS can immediately reveal the role of pleural effusion, soft tissue pathology, lung consolidation or atelectasis as a cause of the physiological diaphane reduction.

Developing techniques related to the lung ultrasound examination has significantly reduced the misdiagnosis rate, improved diagnostic accuracy, and improved the prognosis of infants with correct clinical decision-making (18).

Among congenital abnormalities which LUS can reveal are lung sequestrations and congenital pulmonary airway malformations, but congenital diaphragmatic hernia can also be revealed through this imaging method. Among congenital abnormalities which LUS can reveal are lung sequestrations and congenital pulmonary airway malformations, but congenital diaphragmatic hernia can also be revealed through this imaging method. Neonatal pathologies which can be assessed with LUS include the following ones: transient tachypnea of the newborn (TTN), respiratory distress syndrome (RDS), meconium aspiration syndrome (MAS), bronchopulmonary dysplasia (BPD), and bronchiolitis (19).

The double lung point is a sharp change in echogenicity from highly compact B-lines in the inferior part of the lung and rare B-lines in the upper part. It is a pathognomonic LUS finding of TTN. RDS is characterised by widespread compact B-lines resulting in a white lung pattern, multiple subpleural consolidations and a thickened and irregular pleural line. In preterms, an LUS score based on B-lines, calculated in the first hours of life, can predict the need for intubation for respiratory support and surfactant administration (20, 21). MAS characteristics at LUS are similar to pneumonia but with dynamic modifications over time due to the changing in meconium distribution (22, 23). LUS scores in the first 14 days of life can predict the development of BPD (24). Bronchopulmonary dysplasia (BPD) at LUS shows an irregular pleural line, pleural insect erosion-like change, air vesicle signs, subpleural consolidations and fragment-like echoes, areas of normal lung and B-lines, or even white lung (25, 26). Although LUS is not part of the diagnostic algorithm for bronchiolitis, LUS can monitor the treatment efficacy.

LUS severity scores can predict the clinical course of bronchiolitis and one consolidation >1 cm in the Posterior areas means a relative risk of 4.4 for the need for non-invasive ventilation (27). In bronchiolitis, LUS has high sensitivity and specificity (96.6% and 98.7%, respectively) for identifying patients needing supplemental oxygen (19). In neonatology, in addition to diagnosis, LUS also plays a prominent role in guiding the treatment and following up of lung diseases (28–30). It has also been proposed as a guide to applying for exogenous pulmonary surfactant and ventilation support (31–33). Small handheld portable ultrasound devices allow this imaging technique to be used in remote geographic areas, far away from medical assistance. Specifically, in war scenarios, in the setting of natural disasters or low-income countries with high social inequality, LUS is critical to the diagnosis and management of pediatric patients, leading to ultimately saving lives (34).

## Conclusion

In conclusion, LUS has been rediscovered today thanks to more systematic and evidence-based use. The method demonstrated its usefulness in diagnosis, therapy assistance and following up on various pathological conditions. LUS's cost-effectiveness, innocuity and easy use, together with new technological advancement, allow its use in many different settings. The most promising research fields concern the new applications of thoracic ultrasound and the physical principles underlying the formation of artefacts whose qualitative-quantitative evaluation represents a critical point (3).

The primary limits of this imaging method reside in its well-known operator dependence, which results in possible misuse or uses not supported by adequate training and experience. Therefore, more significant efforts are indispensable by the scientific community in defining the professional profiles of those who can use the method, the necessary training, and the number of procedures to be performed under a tutor's supervision before using the method independently.
